# Study of Organic Honey from the Northeast of Portugal

**DOI:** 10.3390/molecules16075374

**Published:** 2011-06-27

**Authors:** Teresa Gomes, Xesús Feás, Antonio Iglesias, Leticia M. Estevinho

**Affiliations:** 1 CIMO-Mountain Research Center, Agricultural College of Bragança, Polytechnic Institute of Bragança, Campus Santa Apolónia, E 5301-855, Bragança, Portugal; E-Mail: teresa.mdg@gmail.com (T.G.); 2 Department of Analytical Chemistry, Nutrition and Bromatology, University of Santiago de Compostela, E-27002, Lugo, Galicia, Spain; E-Mail: xesusfeas@gmail.com (X.F.); 3 Department of Anatomy and Animal Production, Faculty of Veterinary Medicine, University of Santiago de Compostela, E-27002, Lugo, Galicia, Spain; E-Mail: antonio.iglesias@usc.es (A.I.)

**Keywords:** honey, lavender, organic foods, physicochemical properties, microbiology, phenolic compounds

## Abstract

Concerns about traces of numerous toxic substances and authenticity have prompted consumer demand for honey that is certified as organic, based on strict ecological, natural principles and traceability. The present study aims to characterize organic honey samples (n = 73) from Northeast Portugal, with respect to floral nectar origin, physicochemical parameters and microbial safety. The phenols and flavonoids contents, often referred to as responsible for honey’s bioactive properties, were also assessed. All organic honey samples were classified as monofloral lavender (*Lavandula* sp.), exceeded in quality the international physicochemical standards and showed low microbiological counts (yeast, moulds and aerobic mesophiles), with negative results in respect to fecal coliforms, *Salmonella* and sulphite-reducing *Clostridium* spp. Correlation of the palynological, physicochemical and microbiological results is necessary to check the authenticity, quality and sanitation of honey. Although not required by international legislation, results of those assessments provide a complete outlook and elucidation of the organic honey’s properties, which could promote its valorisation.

## 1. Introduction

Honey is the natural sweet substance produced by honeybees from the nectar of blossoms or from the secretion of living parts of plants or excretions of plant sucking insects on the living parts of plants, which honeybees collect, transform and combine with specific substances of their own, store and leave in the honey comb to ripen and mature [1].

The beneficial characteristics of honey are its high nutritional value and the fast absorption of its carbohydrates on consumption [2,3]. Moreover, when analyzing and studying the therapeutic properties of honeys, modern science has made it possible to specify their medical significance for healing wounds and burns [4], oncology care [5], as well as its antioxidant and antimicrobial factors [6,7,8]. 

Today, having survived all kinds of climatic changes, bees are threatened, and therefore global food security, if pollinators, mainly honeybee, decline or disappear [9]. The cause of the problem is still unknown, which is why it is being described as Colony Collapse Disorder (CCD) and researchers suspect this may be due to a combination of various diseases, environmental pollution, and farming practices, mainly due to an abusive use of increasingly toxic phytosanitary products and large monoculture cropping [10].

In any case, concerns about traces of numerous toxic substances have prompted some consumers to demand honey that is certified as organic [11]. Organic honey production is an ecologically based system, which encourages the use of good agricultural practices to maintain the agricultural ecosystem balance and diversity, promoting the sustainable use of natural resources, environmental quality, animal welfare and human health [12]. Research results indicate that the botanic origin of honey, different beehive types, and the material beehives are made of, all have an influence on the development of bee diseases and the quality of honey [13].

The progressive increase in the market of imported honey, with lower prices and inferior quality, has recently led to a growing need to assess authenticity of local, specially monofloral honeys, using a full quality control based on a physicochemical, microbiological and geographical description [14,15,16,17,18,19].

However the data on full characterization of honey is not abundant and there is a lack of information about the characteristics of honey certified as organic [20].

The present study aims to characterize organic honeys harvested in Northeast Portugal with respect to: (i) floral nectar origin, (ii) physicochemical parameters (moisture, ash, pH, free acidity, electrical conductivity, hydroxymethylfurfural content, apparent sucrose, reducing sugars and diastase activity), (iii) microbial safety (*Salmonella* and sulphite-reducing *Clostridium* spp.), sanitary (fecal coliforms) and commercial quality (aerobic mesophiles, moulds and yeasts) and (iv) phenolic compounds. In this work, and for the first time, the Portuguese organic honeys from five different regions were studied. This might contribute to the valorization of this product, and consequently increase its production.

## 2. Results and Discussion

### 2.1. Pollen Analysis

In Europe more than one hundred botanical species can give monofloral honeys. Most of them have only local importance and are thus marketed on a limited scale, but have also been described in the literature [21]. Moreover, consumers prefer monofloral honeys, which mean that they also have a higher commercial value for the producers. Even though the beekeepers themselves, according to the best of their knowledge and the location of hives, had declared the organic honey samples as monofloral lavender (*Lavandula* sp.) honey, all the samples were subjected to pollen analysis. The identified pollens and their frequency on the analyzed organic honeys are presented on Table 1. Monofloral status generally refers to the presence of a single pollen type in quantities greater than 45% of the total pollen content in the spectrum. For honey samples having under-represented pollen grains, botanical classification may be achieved with a minor pollen frequency percentage, as for example, lavender honey needs 15% of *Lavandula* sp. pollen to be monofloral. Results of the honey’s pollen profile analysis permit us to determine its floral origin and to confirm the identity of the honey source indicated by the beekeepers: the analysed samples had always *Lavandula* sp. as Secondary Pollen (SP, 16-45%) and can thus be classified as monofloral lavender. 

**Table 1:** Frequency classes of pollen types in organic honeys.

	Pollen type							
	*Erica* sp.	*Lavandula* sp.	*Cistus* sp.		*Echium* sp.	*Rubus* sp.		
**Locality**	MP	SP	SP	IMP	SP	SP	IMP	MP
Mogadouro	46.7	100	86.7	13.3	100	100	20	46.7
Milhão	13.3	100	86.7	13.3	100	100	-	9
Angueira	20.0	100	15.0	-	100	100	6.7	80
Bragança	60.0	100	86.7	13.3	100	100	-	80
Vinhais	46.7	100	100	-	100	100	20	33.3
Mean	37.3	100	92.0	8	100	100	9.3	60

SP, Secondary pollen (16 to 44%); IMP, Important minor pollen (3 to 15%) and MP, Minor pollen (1 to 3%).

Other pollens found in the total samples were: *Echium* sp. and *Rubus* sp. (both as 100% of the total honeys as SP), and *Cistus* sp. [present in 92% and 8% of the total honeys as SP and Important Minor Pollen (IMP), respectively]. Next *Prunus* sp. was found as IMP (9.3%) and Minor Pollen (MP, 60%); and *Erica* sp. pollen was found as MP pollen in 37.3% of the total honeys. Variations in nectar content, together with other factors such as climatic conditions, soil type, beekeeper activities and such, contribute to the existence of different types of honeys [22]. Bees forage different plants; thus, honey is always a mixture of several sources. Differences in their composition also mean differences in the organoleptic and nutritional properties of these honeys. Portuguese lavender honeys are generated from the nectar of *Lavandula stoechas*, whereas French lavender honeys are exclusively derived from *Lavandula angustifolia*, *Lavandula latifolia*, or hybrids of these two species [23].

The contribution of the scientific-technical melissopalynology may in the future be useful in commercial transactions, certifying the differentiation of honey for its floral origin, an added value that certainly should not be wasted. Moreover, melissopalynology also allows scientists to infer the vegetation present in an area, and to date and ascertain any biodiversity changes, as for example the presence and distribution of invasive and/or exotic plants. For example, previous work showed that honeys from the Portuguese Entre-Douro e Minho region contained *Eucalyptus* pollen, the latter not being found in the organic lavender honeys analyzed here [19].

### 2.2. Color Analysis

The color of the analyzed samples varied between Light Amber (49 a 83 mm) and Dark Amber (>114 mm), according to the Pfund scale.

### 2.3. Physicochemical Parameters

Table 2 summarizes the results obtained [mean, range and standard deviation (SD)] from the physicochemical analysis of the organic honey samples. The Moisture (M, %) varied from 16.00 to 18.30. The small variation observed in the water contents of these samples may be due to the similar bee-hive handling practices used by Portuguese beekeepers. The M of honey depends on various factors, for example: the harvesting season, the degree of maturity reached in the hive, and environmental factors. The maximum amount of M present in honey is the only composition criterion which, as part of the Honey Standard, has to be met for all world trade honeys. In *Codex Alimentarius* (2001) and EU Council directives (2002) the maximum M content value of pure floral honey is given as 23% for heather honeys and not more than 20% in general. Knowledge of the M contents in honey is useful to improve its conservation and storage by preventing the growth of moulds on its surface. Furthermore, the water content value is also of great importance because it is considered to be a useful parameter for describing moistness and viscosity of honey. Similar results were reported by Gomes *et al.* and by Féas *et al*., in commercial and artisanal honeys from Portugal, respectively.

Hidroxymetilfurfural (HMF) and Diastase Activity (DA) are widely recognized parameters for the evaluation of honey freshness and/or overheating. International regulations set a minimum value of 8 on Gothe’s scale for DA, and a maximum HMF content of 40 mg/kg [1,12]. The HMF content of the honeys analyzed ranged from 0.77 to 1.50 mg/kg. Féas *et al*. and Gomes *et al.* obtained higher values for artisanal (0.2-22.8 mg/kg) and commercial (18.0-94.0 mg/kg) honeys, and as the HMF content is indicative of honey freshness, it suggests that the organic honeys are fresher. This information coincides with the information provided by the beekeepers. The DA of honey samples is 15.35 (Gothe degrees, average) with a range of 13.90 to 16.40 and a SD of 0.61. These amounts are higher than the ones obtained in artisanal (10.30 Gothe degrees) and commercial (12.10 Gothe degrees) honeys. The values obtained in organic honeys for HMF and DA are typical of unprocessed honey. In honey, these parameters are related to its quality and heat processing but have not been related to the origin of the samples [22]. No sample exceeded the limits established for these variables [1,12].

Ash and Electrical Conductivity (EC) values depend on the mineral content of the honey: ash gives a direct measure of inorganic residue after carbonisation, while electric conductivity measures all ionizable organic and inorganic substances. The honeys considered in this study had ash contents ranging from 0.03 to 0.26%. Ash values were below 0.60%, as expected for nectar honeys [1,12]. The ash mass fraction is a useful parameter in determining botanical origin of honey and differentiating between nectar honey and honeydew. The EC values of the honeys analyzed ranged from 0.09 to 0.43 mS/cm. The electrical conductivity of honey may be explained by taking into account the ash and acid content of honey, which reflects the presence of ions and organic acids; the higher their content, the higher the resulting conductivity. The results of ash and EC suggest that the environment isn’t polluted, confirming the high quality of the honeys obtained in biological production mode.

The honey samples presented pH values ranging from 3.45 to 4.00, with an average of 3.74. The observed pH values were similar to those reported for Indian honey samples [24]. The low pH of honey inhibits the presence and growth of microorganisms and makes honey compatible with many food products in terms of pH and acidity. This parameter is of great importance during the extraction and storage of honey as it influences its texture, stability and shelf life. The values of pH in honey help to determine its origin: flower or forest; the latter show higher values. The obtained pH’s values are identical to the referred in studies concerning other trade types of Portuguese honey.

The Free Acidity (FA) of honey samples is 29.8 ± 0.68 meq/kg. Variation in FA among different honeys can be attributed to floral origin or to variation because of the harvest season [25]. The FA of honey may be explained by taking into account the presence of organic acids in equilibrium with their corresponding lactones, or internal esters, and some inorganic ions, such as phosphate. All of the samples investigated met the demands set out in the regulations which require, in general, no more than 50 meq/kg and not more than 80 meq/kg (baker's honey); this indicates the absence of unwanted fermentations [1,12].

Honey is mainly composed of the monosaccharides glucose and fructose. The Reducing Sugars (RS, %) content of the honeys analyzed ranged from 65.60 to 68.90% and the mean percentages of Apparent Sucrose (AS) is 8.01% with a range of 7.50 to 8.30 and a SD of 0.22 (sucrose content by European Directives must be under 5%). These two parameters confirm that the honey samples studied were floral honeys. In respect to reducing sugars (fructose and glucose), EU Directive 2001/110 imposes values ≥60 g/100 g, except for honeydew honey, which is ≥45 g/100 g. These samples do not only meet the standards but also correspond to the levels observed in other studies [6,19,26,27]. Non-reducing sugars (apparent sucrose) are set to be ≤5 g/100 g for the majority of honeys, except for citrus and eucalyptus honeys, which have higher limits (≥10 g/100 g), as well as lavender honeys (≥15 g/100 g). Higher sucrose contents could be the result of an early harvest of honeys, *i.e*., the sucrose has not been converted to fructose and glucose. The sucrose determined for the organic lavender honey (8.01%). In fact, anterior studies carried on by our work team revealed that the sucrose of artisanal and some of the commercial honeys was inferior to the one present in this work. The discrepancy between the sucrose in the different analysed honeys is due to the floral origin, as this study used can only monofloral lavender honeys.

In the analyses of variance of chemical composition variables, the nested term region-sampling location was significant in all cases, suggesting the existence of great variability among the sampling locations within a Portuguese region. In fact, the variability of the chemical composition of honey among sampling locations within a region was higher than the variability among regions, so that on average there were no significant differences among regions, except for Ash and Electrical Conductivity (Table 2). In this case, the Ash and EC from honey produced in Vinhais were significantly lower than the ones verified in the other regions in study.

**Table 2:** Physicochemical parameters of analyzed organic honey samples.

	**Parameters**								
**Locality**	**Moisture (% w/w)**	**Electrical Conductivity (mS/cm)**	**Ash (% w/w)**	**HMF (mg/kg)**	**Diastase Activity (Gothe Scale)**	**pH**	**Free Acidity (meq/kg)**	**Reducing Sugars (%)**	**Apparent Sucrose (%)**
Mogadouro	17.27 ± 0.60 ^a^ (16.50 ± 18.20)	0.25 ± 0.05 ^a^ (0.19 ± 0.33)	0.12 ± 0.07 ^a^ (0.07 ± 0.18)	1.15 ± 0.22 ^a^ (0.89 ± 1.50)	15.30 ± 0.71 ^a^ (14.00 ± 16.20)	3.76 ± 0.13 ^a^ (3.60 ± 4.00)	40.48 ± 0.56 ^a^ (39.70 ± 41.20)	67.78 ± 0.45 ^a^ (66.90 ± 68.30)	7.94 ± 0.20 ^a^ (7.60 ± 8.25)
Milhão	17.17 ± 0.58 ^a^ (16.50 ± 18.20)	0.32 ± 0.08 ^a^ (0.20 ± 0.43)	0.17 ± 0.10 ^a^ (0.07 ± 0.26)	1.22 ± 0.17 ^a^ (0.97 ± 1.50)	15.48 ± 0.38 ^a^ (14.90 ± 16.00)	3.69 ± 0.08 ^a^ (3.50 ± 4.00)	40.24 ± 0.68 ^a^ (39.40 ± 41.30)	67.85 ± 0.18 ^a^ (67.40 ± 68.30)	8.06 ± 0.16 ^a^ (7.75 ± 8.20)
Angueira	17.21 ± 0.68 ^a^ (16.40 ± 18.30)	0.33 ± 0.04 ^a^ (0.29 ± 0.40)	0.18 ± 0.04 ^a^ (0.15 ± 0.24)	1.08 ± 0.09 ^a^ (0.93 ± 1.20)	15.57 ± 0.3 ^a^ (14.99 ± 16.20)	3.80 ± 0.09 ^a^ (3.65 ± 4.00)	40.32 ± 0.81 ^a^ (38.90 ± 41.20)	67.91 ± 0.67 ^a^ (65.90 ± 68.90)	8.21 ± 0.11 ^a^ (7.89 ± 8.30)
Brangança	17.24 ± 0.57 ^a^ (16.50 ± 18.10)	0.26 ± 0.09 ^a^ (0.18 ± 0.43)	0.12 ± 0.11 ^a^ (0.06 ± 0.26)	1.17 ± 0.2 ^a^ (0.87 ± 1.50)	15.18 ± 0.6 ^a^ (14.00 ± 16.20)	3.79 ± 0.14 ^a^ (3.60 ± 3.99)	40.51 ± 0.63 ^a^ (39.50 ± 41.30)	67.91 ± 0.38 ^a^ (67.10 ± 68.30)	7.96 ± 0.22 ^a^ (7.60 ± 8.30)
Vinhais	16.97 ± 0.67 ^a^ (16.00 ± 18.00)	0.13 ± 0.05 ^b^ (0.19 ± 0.25)	0.06 ± 0.01 ^b^ (0.03 ± 0.12)	1.00 ± 0.22 ^a^ (0.77 ± 1.35)	15.21 ± 0.81 ^a^ (13.90 ± 16.40)	3.67 ± 0.15 ^a^ (3.45 ± 3.85)	40.01 ± 0.64 ^a^ (38.90 ± 41.20)	67.54 ± 0.75 ^a^ (65.60 ± 68.50)	7.88 ± 0.23 ^a^ (7.50 ± 8.15)
Mean	17.17 ± 0.62 (16.00 ± 18.30)	0.26 ± 0.09 (0.09 ± 0.43)	0.13 ± 0.05 (0.07 ± 0.26)	1.14 ± 0.20 (0.77 ± 1.50)	15.35 ± 0.61 (13.90 ± 16.40)	3.74 ± 0.13 (3.45 ± 4.00)	40.31 ± 0.68 (38.90 ± 41.30)	67.80 ± 0.53 (65.60 ± 68.90)	8.01 ± 0.22 (7.50 ± 8.30)

* The letters (a and b) represents which honeys are different by Tukey test with significance of p = 0.05.

### 2.4. Bioactive Compounds

The total phenols of the analyzed honey varied from 170 to 239 mg/Kg. The average flavonoids’ value of the honey samples is 124.70 mg/Kg with a range of 117.00 to 135.00 mg/Kg and a SD of 15.7 (Table 3).

**Table 3:** Differences in phenols and flavonoids mean concentrations among regions. Standard deviation values are shown in brackets.

	Parameters	
Locality	Phenols (mg/Kg)	Flavonoids (mg/Kg)
Mogadouro	198.50 ± 8.9 ^c^ (170.20 ± 238.30)	135.00 ± 24.9 ^a^ (117.30-176.50)
Milhão	201.20 ± 26.40 ^b c^ (187.50 ± 202.70)	117.00 ± 8.9 ^c^ (109.60 ± 125.30)
Angueira	201.90 ± 45.5 ^b c^ (189.90 ± 234.10)	123.70 ± 6.60 ^b c^ (117.80 ± 126.30)
Bragança	214.30 ± 20.9 ^a^ (187.50 ± 239.50)	126.40 ± 17.80 ^b^ (116.90 ± 167.40)
Vinhais	212.90 ± 9.0 ^a b^ (198.90 ± 225,60)	121.50 ± 6.8 ^b c^ (117.10 ± 124.10)
Mean	205.70 ± 26.2	124.70 ± 15.70

* The letters (a, b and c) represents which honeys are different by Tukey test with significance of p = 0.05.

The obtained results both for total phenols and for flavonoids were identical to the ones reported by our work team in light honeys from the Parque Natural de Montesinho [28].

### 2.5. Microbiological Analyses

Microbial counts in organic honey samples are presented in Table 4. Honey, in spite of its usefulness, is known to contain certain microbes. The microorganisms that survive in honey are those that withstand the concentrated sugar, acidity and other antimicrobial characters of honey. In any case, this natural reservoir for microbes’ status does not diminish the many important uses that honey is known for. 

Levels of quantification for the commercial quality parameters (aerobic mesophiles and moulds and yeasts) in the analyzed honey samples are generally lower than those reported by Féas *et al*. Yeast and moulds were detected in low counts, with means values obtained of 14.1 ± 4.5 cfu/g. The total aerobic mesophiles counts in the samples ranged from 1.9 × 10^2^ cfu/g to 2.1 × 10^2^ cfu/g, with a mean value obtained of 2.0 × 10^2^ cfu/g ± 3.2 × 10^1^ cfu/g. These results are similar to the determinated by Gomes *et al.* in commercial honeys.

It was found that the most important variables greatly associated to the levels of mesophiles and moulds in honey were in this order the levels of moisture, ash (related to solids), acidity and HMF (p < 0.001) (results not shown). From the microbiological point of view, these low values of moulds and yeasts would be most probably related to environmental conditions, and are indicative of an appropriate management of organic apiaries. 

**Table 4:** Microbial analyses of organic honey samples.

	Locality				
Microorganisms	Mogadouro	Milhão	Angueira	Bragança	Vinhais
Moulds and Yeasts ^a^	1.5 × 10 ± 5	1.6 × 10 ± 5	1.2 × 10 ± 3	1.5 × 10 ± 4	1.5 × 10 ± 4
Aerobic mesophiles ^a^	1.9 × 10^2^ ± 4.3 × 10^1^	2.1 × 10^2^ ± 3.9 × 10^1^	1.9 × 10^2^ ± 2.4 × 10^1^	1.9 × 10^2^ ± 2.5 × 10^1^	1.9 × 10^2^ ± 2.5 × 10^1^
Fecal coliforms ^b^	± 1	± 1	± 1	± 1	± 1
Sulphite-reducing *Clostridium* spp. ^c^	-	-	-	-	-
*Salmonella* ^d^	-	-	-	-	-

^a^ Colony-forming units per gram of honey (cfu/g). ^b^ Fecal coliforms were enumerated by the Most Probable Number (MPN). ^c^ in 0.01 g. ^d^ in 25 g.

In respect to sanitary quality (fecal coliforms) and safety (sulphite-reducing *Clostridium* spp. and *Salmonella*), all our samples were negative, as verified by our team in other Portuguese honeys. In contrast, other authors detected coliform contamination in one tested sample [29], and reported that 70% of 23 honey samples were contaminated with sulphite-reducing *Clostridium* spp. [30]. Having reviewed the scientific literature, *Clostridiums* appears to be the main microorganism in honey of concern to human health. Its presence in honey is especially dangerous for babies under one year old, since they do not have a fully developed immune system, and honey is the only dietary vehicle so far definitively linked to infant botulism. Honey samples collected at retail level were found to be contaminated with *Clostridium botulinum* spores in the USA (10% of the analysed samples), Japan (8.5%), Brazil (7.5%) and Italy (6.5%); with contamination levels between 5 and 80 spores g^−1^ of product [31]. EU legislation lacks specifications concerning microbiological contamination and hygiene of the product. In fact, numerous studies have been reported on the physicochemical parameters of honeys from all over the world, but microbiological contamination studies are few and are essentially devoted to *C. botulinum*. It is recommended that effective and targeted information regarding risks of infant botulism from the consumption of honey should be provided, e.g. *via* leaflets, labelling and advice to health-care professionals.

## 3. Conclusions

The present study has characterised organic honey harvested in Northeast Portugal, in respect to floral origin, physicochemical parameters, microbial safety and bioactive compounds. All organic honey samples can be classified as monofloral lavender and all the values obtained for the physicochemical and microbiological parameters exceed in quality the limits imposed by the present legislation. These results show that organic honey production, with the current beekeeping handling and extraction applied by Portuguese beekeepers, fulfil all the required quality and safety parameters.

Despite the quality of the organic honey, further trials are required, as significant differences between this product and other honeys (commercialised and artisanal) from the North and Centre of Portugal were observed. Those studies should include the evaluation of the botanical origin of honey by amino acids content.

## 4. Experimental

### 4.1. Honey Sampling

Seventy-three (n = 73) organic honey samples, from *Apis mellifera*, were collected from July to August in 2009 by twenty five beekeepers from separate apiaries. The samples were from five localities of Northeast Portugal with the following distribution: Mogadouro (n = 16); Milhão (n = 15); Angueira (n = 13); Bragança (n = 15) and Vinhais (n = 14). They were obtained by centrifugation and stored at 5 °C until analysis, which occurred no more than one month after the extraction from the hives by beekeepers. 

### 4.2. Reagents and Equipment

Gallic acid, (+)-catechin and ethanol were obtained from Sigma-Aldrich (Germany). Methanol was obtained from Pronolab (Lisboa, Portugal). The Folin-Ciocalteu and sodium carbonate reagents were obtained from Merck (Germany). All other chemicals were obtained from Sigma Chemical Co. (St. Louis, USA). All the chemicals used in the experiments were of analytical grade. Water was treated in a Mili-Q Water Purification System (TGI Pure Water System, USA). The spectrophotomer was a model Unicam UV-Visible Spectrometry Hekios (United Kingdom).

### 4.3. Sample Floral-Type Identification

Even though the beekeepers themselves declared honey as monofloral lavender, all the samples were subjected to pollen analysis *per* the acetolysis method of Erdtman as reported previously in detail [19]. The following terms were used for pollen frequency classes: predominant pollen (PP, more than 45% of pollen grains counted), secondary pollen (SP, 16-45%), important minor pollen (IMP, 3-15%) and minor pollen (MP, 1-3%).

### 4.4. Color Analysis

Honey samples were heated to 50 °C to dissolve sugar crystals, and the colour was determined by spectrophotometric (Unicam UV-Visible Spectrometry) measurement of the absorbance of a 50% honey solution (w/v) at 635 nm. The honeys were classified accordingto the Pfund scale after conversion of the absorbance values: mm Pfund = 38.70 + 371.39 × Abs [32]. 

### 4.5. Physicochemical Analysis

Analyses of the physicochemical properties of organic honey samples were performed in accordance with The *Official Methods of Analysis* of the Association of Official Analytical Chemists, reported previously in detail [17]. Three replicate analyses were made from each sample to obtain the reported data. The evaluated parameters were: water content (M, g per 100 g), ash (g per 100 g), electrical conductivity (EC, mS/cm), hydroxymethylfurfural content (HMF, mg/kg), free acidity (FA, meq/kg), diastase activity (DA, Gothe degrees), reducing sugars (RS, g per 100 g), apparent sucrose (AS, g per 100 g) and pH. 

### 4.6. Microbiological Determinations

Ten grams of each honey sample were homogenized into peptone water solvent (90 mL). Decimal dilutions were made into the same solvent. Aerobic mesophilic bacteria were counted onto standard Plate Count Agar (PCA, Hemidea) and incubated at 30 °C for 48 h (NP-3788:2002). Microbial counts were expressed as colony-forming units per gram of bee pollen (cfu/g). Moulds and yeasts enumeration was made on DG18 (Himedia) incubated at 25 °C for 5 days, following the ISO 21527-2 (2008) protocol. Microbial counts were expressed as colony-forming units per gram of bee pollen (cfu/g).

For sulphite-reducing *Clostridium* spp. counting, aliquots of 10, *5*, 1 and 0.1 mL of the initial suspension were added to an empty tube, thermally treated at 80 °C for 5 min and covered with Iron Sulfite Agar (ISA) media (ISO 15213:2003), tubes were incubated at 37 °C for 5 days. Fecal coliforms were enumerated by the Most Probable Number technique defined in the protocol ISO 4831 (2006). Results were expressed as most probable numbers of coliforms per gram of bee pollen (MPN/g). The positive results for fecal coliforms were researched for *E. coli*. Enumeration was made on Eosin Methylene Blue Agar-EMB Agar (Oxoid), incubated at 35 °C for 24 h. Results were expressed as colony forming units of *E. coli* per gram of bee pollen (cfu/g). *Salmonella* detection followed the ISO 6579 protocol. All microbial tests were performed in triplicate.

### 4.7. Bioactive Compounds Quantification

The total phenolic content was estimated according to the Folin-Ciocalteau method described by Moreira *et al*. The reaction of MEP (0.5 mL) mixed with the Folin-Ciocalteau reagent (0.5 mL) and 0.5 mL of 10% Na_2_CO_3_ was kept in the dark at room temperature for 1 h, and the absorbance was quantified at 700 nm (Unicam UV-Visible Spectrometry) acid was used to calculate the standard curve (0.01-0.08 mM; y = 2.3725x + 0.0021; R2 = 0.1). Total phenols content were expressed as mg of Gallic acid equivalents/g of extract (GAEs). 

Flavonoids were determined according to procedures described by [33]. The sample (250 µL) was mixed with distilled water (1.25 mL) and 5% NaNO_2_ solution (75 µL). After 5 min, a 10% AlCl_3_·H_2_O (150 µL) solution was added. After 6 min, 1 M NaOH (500 µL) and distilled water (275 µL) were added to the mixture. The solution was mixed well and the intensity of pink colour was measured at 510 nm. (+)-Catechin was used to calculate the standard curve (0.022-0.34 mM; Y = 0.9990X − 0.0497; R2 = 0.9961) and the results were expressed as mg of (+)-catechin equivalents (CEs) per kg of honey.

As the reducing sugars, which are present in large amounts in honey have been found to interfere with the original Folin-Ciocaltu metod, in order to avoid overestimated results, a blank was prepared with honey extract (1:2 honey/water) and insoluble polyvinylpolypyrrolidine (PVPP, 0.1 g) as proposed by Gheldof *et al.*

### 4.8. Statistical Analysis

Each honey was analyzed in triplicate. Results are shown as mean values and standard deviation. In each variable, the differences between region were analyzed using one-way analysis of variance (ANOVA) followed by Tukey’s HSD Test with α = 0.05. This treatment was carried out using SPSS 18.0 for Windows^® ^Program.
